# Neoplastic risk in hyperplastic esophagogastric junction lesions: Comprehensive multicenter study

**DOI:** 10.1055/a-2760-6753

**Published:** 2025-12-16

**Authors:** Elena De Cristofaro, Federico Barbaro, Jérôme Rivory, Alexandru Lupu, Benedetto Neri, Dario Biasutto, Gianluca Andrisani, Rui Morais, Franscisco Mendes, João Santos-Antunes, Germana de Nucci, Sandro Sferrazza, Silvia Pecere, Yanis Dahel, Jean-Philippe Ratone, Laura Rovedatti, Cristiano Spada, Francesco Maria Di Matteo, Andrea Anderloni, Philippe Leclercq, Samanta Romeo, Guido Manfredi, Elisa Stasi, Jeremie Jacques, Edoardo Troncone, Lucile Héroin, Jerome Maitre, Arthur Berger, Giulio Antonelli, Simona Agazzi, Giovanna Del Vecchio Blanco, Giovanni Monteleone, Mathieu Pioche

**Affiliations:** 1 60259 Faculty of Medicine and Surgery, Gastroenterology, University of Rome Tor Vergata, Rome, Italy; 2 18654 Digestive Endoscopy Unit, Fondazione Policlinico Universitario Agostino Gemelli IRCCS, Roma, Italy; 3 Gastroenterology, Edouard Herriot Hospital, Lyon, France; 4 Gastroenterology and Endoscopy, Pavillon L Edouard Herriot Hospital, Lyon, France; 5 9318 Department of Systems Medicine, University of Rome Tor Vergata, Roma, Italy; 6 220431 Therapeutic GI Endoscopy Unit, Campus Bio-Medico University Hospital, Roma, Italy; 7 220431 Digestive Endoscopy Unit, Campus Bio-Medico University Hospital, Roma, Italy; 8 467113 Gastroenterology, Hospital São João, Porto, Portugal; 9 70918 Glycobiology and Cancer, IPATIMUP, Porto, Portugal; 10 Gastroenterology and Endoscopy Unit, A.O. Salvini, Garbagnate Milanese (MI), Italy; 11 367405 Gastroenterology Unit, Department of Medical and Surgical Sciences, ARNAS Civico Di Cristina Benfratelli, Palermo, Italy; 12 Digestive Endoscopy Unit, Catholic University, Gemelli University Hospital, Rome, Italy; 13 Endoscopy Unit, Paoli Calmettes Institute, Marseille, France; 14 Gastroenterology, Paoli-Camettes, Marseille, France; 15 19001 First Department of Internal Medicine, Fondazione IRCCS Policlinico San Matteo, University of Pavia, Pavia, Italy; 16 220431 Operative Digestive Endoscopy Department, Campus Bio-Medico University Hospital, Roma, Italy; 17 18631 Endoscopy, Fondazione IRCCS Policlinico San Matteo, Pavia, Italy; 18 60182 Gastroenterology and Hepatology, University Hospitals Leuven, Leuven, Belgium; 19 18550 Gastroenterology and Endoscopy Department, Maggiore Hospital Crema, Crema, Italy; 20 Gastroenterology, Digestive Endoscopy, Vito Fazzi Hospital, Lecce, Italy; 21 Service d'Hépato-Gastro-Entérologie, CHU Dupuytren Limoges, Limoges, France; 22 9318 Department of Systems Medicine, University of Rome Tor Vergata, Rome, Italy; 23 36604 Gastroenterology and Hepatology, University Hospitals Strasbourg, Strasbourg, France; 24 36604 Gastroenterology and Digestive Endoscopy Unit, Strasbourg University Hospitals, Strasbourg, France; 25 36836 Pôle Hépato-Gastro-Entérologie, Diabétologie, Nutrition et Endocrinologie, Centre Hospitalier Universitaire de Bordeaux, Bordeaux, France; 26 638740 Gastroenterology and Endoscopy Unit, Ospedale dei Castelli, Rome, Italy; 27 18631 Gastroenterology and Digestive Endoscopy, Fondazione IRCCS Policlinico San Matteo, Pavia, Italy; 28 9318 Gastroenterology Unit, University of Rome Tor Vergata, Rome, Italy; 29 Department of Systems Medicine, University of Rome Tor Vergata, Rome, Italy

**Keywords:** Endoscopy Upper GI Tract, Endoscopic resection (ESD, EMRc, ...), Precancerous conditions & cancerous lesions (displasia and cancer) stomach, Diagnosis and imaging (inc chromoendoscopy, NBI, iSCAN, FICE, CLE)

## Abstract

**Background and study aims:**

Esophagogastric junction (EGJ) lesions are uncommon and histologically diverse. Among these, EGJ hyperplastic lesions are rare and generally considered benign. However, their nonspecific appearance makes accurate endoscopic identification challenging. Endoscopic resection is both a diagnostic and therapeutic approach, yet risk factors for neoplastic transformation in EGJ lesions remain unclear. This study aimed to identify predictive factors for neoplastic transformation in hyperplastic EGJ lesions.

**Patients and methods:**

This multicenter, retrospective study included patients with hyperplastic EGJ lesions endoscopically resected across 13 European hospitals. Data were collected from endoscopy and pathology reports. Neoplastic transformation was defined by presence of dysplasia or adenocarcinoma. A multivariable logistic regression model was conducted to assess predictive factors for neoplastic transformation in resected hyperplastic lesions.

**Results:**

From January 2015 to October 2024, 91 EGJ hyperplastic lesions were included. Polypectomy/endoscopic mucosal resection (EMR) was performed in 86% of cases, endoscopic submucosal dissection (ESD) in 19%. En bloc resection was successfully achieved in 93% of cases, whereas R0 resection rates were confirmed in 84% of cases. Twenty-one lesions (23%) showed neoplastic transformation on histology. Independent predictive factors for neoplastic transformation in hyperplastic lesions included non-polypoid morphology (odds ratio [OR] 5.48; *P* = 0.025), presence of surface ulceration (OR 11.5; *P* = 0.0005) and lesion size (OR 5.48; *P* = 0.021). Lesion size > 12 mm was identified as a significant predictor of neoplastic transformation in hyperplastic lesions.

**Conclusions:**

EGJ hyperplastic lesions showed a non-negligible risk of neoplastic transformation. These findings highlight the need for careful endoscopic assessment to predict malignancy while promoting appropriate management strategies to ensure adequate R0 resection in case of undetected local malignancy.

## Introduction

Esophagogastric junction (EGJ) lesions represent a distinct and uncommon subset of gastrointestinal lesions, encompassing a heterogeneous group of histopathologic entities. These include adenoma, squamous papilloma, leiomyoma, Barrett's esophagus (BE)-associated polypoid dysplasia, polypoid carcinoma, inflammatory fibroid polyps, and fundic gland polyps 1 2 3 . Hyperplastic lesions of the EGJ are another rare type of polyp, characterized by proliferation of gastric-type foveolar epithelium, squamous epithelium, or both 4 5 .

The first systematic analysis of hyperplastic EGJ polyps was conducted in the early 2000s by Abraham et al. 6 , who reported that these lesions were most often located at the EGJ (67%), followed by the distal esophagus (30%). Most polyps (80%) were predominantly composed of cardiac-type mucosa, with a smaller proportion consisting of squamous mucosa (17%) or a mixture of both (3%). Intestinal metaplasia and low-grade dysplasia were rare, observed in 7% and 3% of cases, respectively. However, this study analyzed only 30 hyperplastic polyps from 27 patients and did not fully elucidate endoscopic features of EGJ lesions.

Differentiating hyperplastic polyps of the EGJ from other lesions in this region remains challenging, due to their nonspecific macroscopic appearance and limited understanding of their neoplastic potential 7 8 9 . Although some studies suggest a potential association with BE and gastroesophageal reflux disease (GERD) 5 10 11 , the evidence is conflicting. In addition, unlike other gastric locations, the relationship between EGJ hyperplastic lesions and *Helicobacter pylori* infection remains unclear 6 12 . Overall, prevalence of dysplasia arising in hyperplastic lesions remains debated, with reported rates ranging from 1.9% to 19% 13 14 15 16 17 . Similarly, risk of adenocarcinoma has been documented in larger studies, varying from 0% to 13.5% 13 15 16 18 19 20 . This wide variability complicates clinical decision-making regarding need for resection and long-term surveillance. Moreover, limited data are available specifically on EGJ lesions and their potential risk of malignant transformation. Understanding the neoplastic potential of these lesions is crucial for guiding endoscopic management and surveillance strategies. This study aimed to assess risk of neoplastic transformation in hyperplastic EGJ lesions.

## Patients and methods

### Study group

This retrospective multicenter study investigated EGJ hyperplastic lesions across 13 European hospitals, including three centers in France, one in Portugal, one in Belgium, and eight in Italy, between January 2015 and October 2024. Patients were identified retrospectively using disease coding systems or prospectively maintained databases at each center. Relevant clinical data were extracted from endoscopy, pathology, and hospitalization reports and anonymized prior to analysis.

Inclusion criteria comprised patients aged ≥ 18 years who underwent endoscopic resection of a hyperplastic lesion located at the EGJ. Exclusion criteria included lesions recurring at the site of a previous endoscopic resection, non-hyperplastic lesions, hyperplastic lesions not resected, and patients with hereditary gastric polyposis syndromes (e.g., familial adenomatous polyposis or hamartomatous polyposis). Additional clinical variables were recorded, including presence of BE, history of GERD—defined as presence of typical symptoms such as heartburn and/or regurgitation at least twice per week 21 —and *Helicobacter pylori* infection. Endoscopic follow-up data were collected when available. Lesions were classified according to the Paris classification 22 23 as polypoid (sessile 0-Is, pedunculated 0-Ip, or semi-pedunculated 0-Isp) or non-polypoid (slightly elevated 0-IIa, flat 0-IIb, or slightly depressed 0-IIc). Anatomical location was further categorized into greater or lesser curvature and anterior or posterior wall. Vascular and pit patterns were described as regular or irregular and presence of surface ulceration was noted.

Resection technique was categorized as endoscopic mucosal resection (EMR)/polypectomy, endoscopic submucosal dissection [ESD]), or other. Immediate and delayed adverse events, such as bleeding, perforation, or stenosis, were identified retrospectively from medical records and classified according to the Adverse events in GastRointEstinal Endoscopy (AGREE) system 24 .

### Definitions

EGJ lesions were defined as suspected hyperplastic lesions located within 2 cm above or below the endoscopic EGJ. Histologically, they were classified as hyperplastic polyps based on presence of elongated, tortuous, and dilated foveolar glands lined by mucin-rich epithelium, often associated with surface erosions and a chronically inflamed lamina propria. En bloc resection was defined as complete removal of the lesion in a single piece without fragmentation. R0 resection was defined as en bloc resection with tumor-free horizontal and vertical margins. Neoplastic transformation was defined by histological presence of dysplasia or adenocarcinoma arising within a hyperplastic EGJ lesion 25 .

### Objectives

The primary objective was to identify independent predictive factors associated with neoplastic transformation in EGJ hyperplastic lesions that underwent endoscopic resection.

Secondary objectives included assessment of recurrence rates following endoscopic resection and evaluation of en bloc and R0 resection rates according to the technique used.

### Statistical analysis

Quantitative variables were described using the mean and standard deviation or the median with the range and interquartile range (IQR). Qualitative variables were summarized by frequency and percentage, excluding missing data from percentage calculations. The effect of various factors on risk of neoplastic transformation was quantified using odds ratios (ORs) with corresponding 95% confidence intervals (CIs). Group comparisons were conducted using Student’s *t* -test, chi-squared test, or Mann-Whitney U test (Wilcoxon rank-sum test), depending on data distribution.

Univariable analyses were performed using mixed logistic regressions to explore associations between potential risk factors and neoplastic transformation. Variables with *P* < 0.01 in univariable analysis were then included in a multivariable logistic regression model, followed by a backward selection process to identify the most significant predictors. *P* < 0.05 was considered statistically significant. The receiver operating characteristic (ROC) curve was plotted to determine the optimal cut-off point for lesion size as a predictor of neoplastic transformation. Statistical analyses were performed on all available data (SPSS 29.0).

### Ethical considerations

The study was conducted according to the Declaration of Helsinki and received approval from the ethics committee of the Policlinico Tor Vergata (Rome), February 28, 2024, code 15.24 CET2 ptv.

## Results

### Study populations and outcomes

**Fig. 1:**
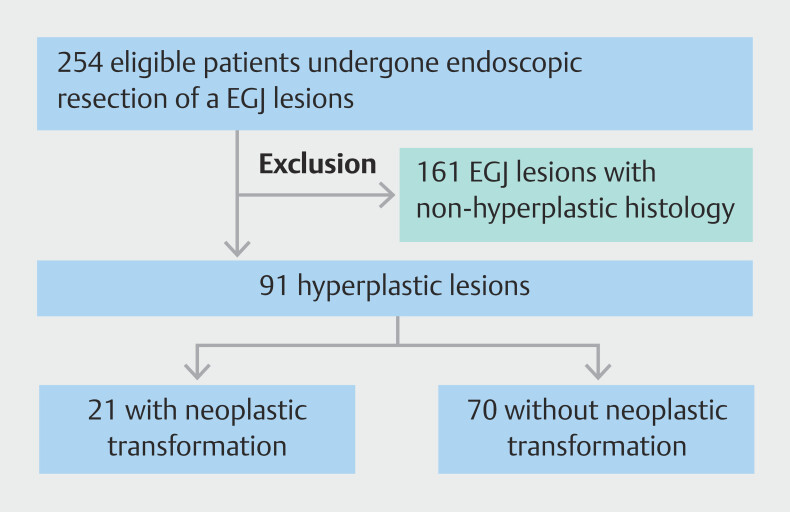
Flowchart of the study.

Between January 2015 and October 2024, a total of 254 endoscopically resected EGJ lesions were recorded. After excluding non-hyperplastic lesions, 91 EGJ hyperplastic lesions were included in the study ( Fig. 1 ). The clinical and endoscopic characteristics of cohort are presented in Table 1 .

**Table:** **Table 1** Clinical and endoscopic characteristics of full cohort (91 patients).

**Characteristics (91 pts)**	
Age at endoscopy; Median (IQR, range)	72 (21.0; 20–97)
Sex, n (%)	
Male	45(47)
Female	48 (53)
Barrett’s esophagus, n (%)	
Yes	7 (8)
No	83 (91)
Missing data	1 (1)
History of esophageal reflux disease	
Yes	28 (31)
No	48 (53)
Missing data	15 (16)
Helicobacter pylori infection, n (%)	
Yes	4 (4)
No	66 (73)
Missing data	21 (23)
Polyp size	
Median IQR, (range)	10 (12.0; 3–60)
< 25 mm, n (%)	76 (83.5)
≥ 25 mm, n (%)	15 (16.5)
Location, n (%)	
Greater curvature, anterior wall	21 (23)
Greater curvature, posterior wall	22 (24)
Lesser curvature, anterior wall	11 (12)
Lesser curvature, posterior wall	11 (12)
Missing data	26 (29)
Morphology, Paris Classification n (%)	
0-Is	51 (56)
0-Isp/0-Ip	20 (22)
0-IIa	10 (11)
0-IIb	2 (2)
0-IIc	2 (2)
Missing data	6 (7)
Pit Pattern, n (%)	
Regular	72 (79)
Irregular	6 (7)
Missing data	13 (14)
Presence of surface ulceration, n (%)	
Yes	19 (21)
No	61 (67)
Missing data	11 (12)
Type of resection, n (%)	
EMR/polypectomy	78 (86)
ESD	17 (19)
Others [cap-mucosectomy)	3 (3)
En bloc resection, n (%)	
Yes	85 (93)
No	6 (7)
Complications, n (%)	
Bleeding	11 (12)
Stenosis	1 (0.8)
Quality of resection, n (%)	
R0	76 (84)
R1	15 (16)
IQR, interquartile range.	

The cohort predominantly consisted of elderly patients (72 years; IQR 21.0), with a slight male predominance (47%). A minority had a history of GERD (31%) or BE (8%). *Helicobacter pylori* status was available for 70 out of 91 patients, with four positive cases (6%).

Median lesion size was 10 mm (IQR 12.0; range: 3–60 mm). Seventy-seven of 91 lesions (85%) were classified as polypoid, whereas 14 lesions (15%) were non-polypoid according to the Paris classification. Vascular and pit patterns were described as regular in 71 (78%) and 72 (79%) lesions, respectively. Surface ulceration was observed in 19 cases (21%).

Of 91 evaluable lesions, 78 (86%) were resected using conventional EMR, 17 (19%) with ESD, and three (3%) with cap-assisted EMR. En bloc resection was achieved in 85 cases (93%) and R0 resection was confirmed in 76 lesions (84%), including 60 of 78 (77%) after EMR and 16 of 17 (94%) after ESD. Immediate post-procedure bleeding occurred in 11 cases (12%) and was managed endoscopically. One patient developed a stricture after resection of a large (45 mm), nearly circumferential lesion.

### Recurrence after endoscopic resection

Among the 91 patients, 38 (42%) underwent at least one endoscopic follow-up, with the first control endoscopy performed after a median interval of 7 months. During follow-up, 10 of 38 patients (26%) presented with recurrence of hyperplastic lesions. Notably, eight of 10 recurrences occurred in lesions initially resected using EMR, whereas two recurrences were observed in lesions resected by ESD, despite histologically confirmed R0 resection. All recurrent lesions were successfully managed with repeat endoscopic resection.

### Predictive factors of neoplastic transformation in hyperplastic lesions

**Fig. 2:**
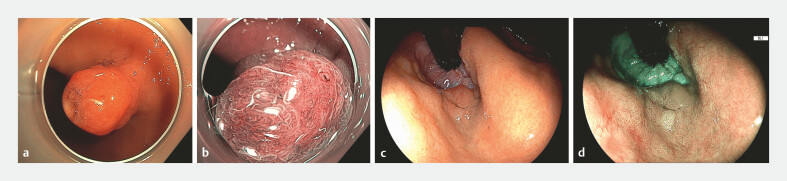
Representative examples of hyperplastic lesions of the esophagogastric junction. **a** Malignant hyperplastic lesion showing a distinct neoplastic component under white light imaging. **b** The same lesion observed in close-up view with dual focus and narrow-band imaging, demonstrating irregular microsurface and microvascular patterns consistent with neoplastic transformation. **c** Non-dysplastic hyperplastic polyp of the esophagogastric junction, visualized in retroflexed view under white light. **d** The same lesion examined with blue light imaging (BLI).

Histological examination revealed that 21 of 91 hyperplastic lesions (23%) exhibited neoplastic transformation. Among these, seven were classified as low-grade dysplasia, two as high-grade dysplasia, and 12 as adenocarcinoma (including 10 intramucosal and 2 with submucosal invasion). An example of a malignant hyperplastic lesion is presented in Fig. 2 . Median lesion size was 14 mm (IQR 28; 12–40) for dysplastic lesions and 10 mm (IQR 10 mm; 8–18) for adenocarcinomas. Surface ulceration was present in one of nine (11%) dysplastic lesions and nine of 12 (75%) were adenocarcinomas.

Univariable analysis identified surface ulceration (OR 5.05, 95% CI 1.67–15.35; *p* = 0.004), non-polypoid morphology (OR 3.81, 95% CI 1.18–12.27; *P* = 0.027), and lesion size (OR 1.04, 95% CI 1.01–1.09; *p* = 0.033) as significant risk factors for neoplastic transformation.

These associations were confirmed in multivariable analysis, which demonstrated that surface ulceration (OR 11.5, 95% CI 2.88–45.78; *P* = 0.0005), non-polypoid morphology (OR 5.48, 95% CI 1.29–23.19; *P* = 0.025), and lesion size (OR 1.06, 95% CI 1.01–1.11; *P* = 0.021) were independent predictors of dysplasia ( Table 2 ).

**Table:** **Table 2** Univariable and multivariable analysis to identify factors predictive of neoplastic transformation in hyperplastic esophagogastric junction lesions.

	**Univariable analysis**		**Multivariable analysis**	
	**OR (95% CI)**	***P* value **	**OR (95% CI)**	***P* value **
Greater curvature location Ref: Lesser curvature	1.66 (0.55–5.02)	0.37		
Size, median (range)	1.04 (1.01–1.09)	**0.033**	5.48 (1.01–1.11)	**0.021**
Non polypoid morphology Ref: Polypoid	3.81 (1.18–12.3)	**0.027**	5.48 (1.29–23.2)	**0.025**
Irregular pit pattern Ref: Regular	3.23 (0.59 17.5)	0.18		
Irregular vascular pattern Ref: Regular	1.96 (0.31–12.7)	0.49		
Presence of ulcerate surface	5.05 (1.67–15.3)	**0.004**	11.5 (2.88–45.8)	**0.0005**
Barrett’s esophagus	1.12 (0.2 – 6.05)	0.89		
*Helicobacter pylori* infection	054 (0.05–4.98)	0.57		
OR, odds ratio.				

**Fig. 3:**
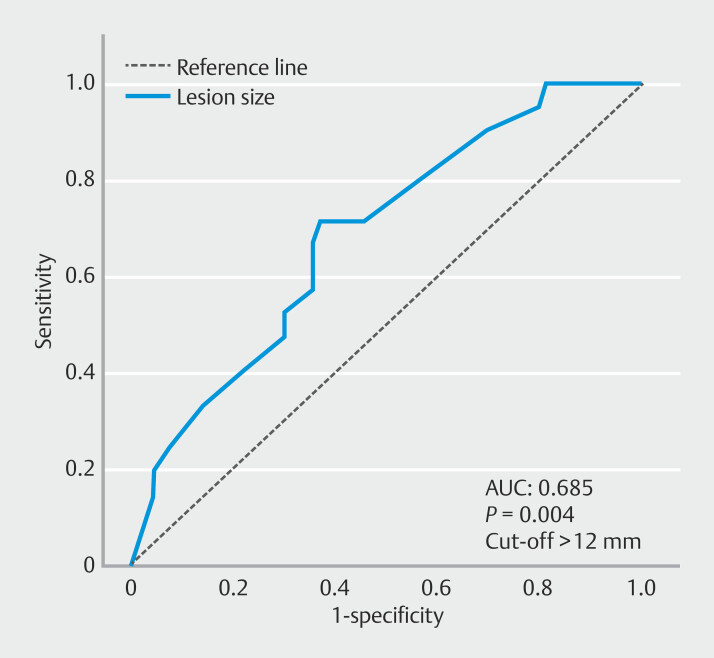
Receiver operating curve for neoplastic transformation based on the lesion size.

The predictive value of lesion size for neoplastic transformation, including dysplastic lesions and adenocarcinomas, was further evaluated using ROC curve analysis. The area under the ROC curve was 0.685 (95% CI: 0.561–0.809; *P* = 0.004). The optimal cut-off value was 12 mm, yielding a sensitivity of 71% and a specificity of 63% ( Fig. 3 ).

## Discussion

This multicenter study evaluated risk of neoplastic transformation in EGJ hyperplastic lesions, a condition traditionally considered rare and benign 26 , and therefore, often left unresected. However, the mechanisms underlying potential malignant transformation and the associated risk factors remain poorly defined. The previous analysis, conducted by Abraham et al. 6 , included a limited cohort of 30 polyps from 27 patients and did not provide a comprehensive characterization of EGJ lesions, particularly regarding their endoscopic features and neoplastic potential.

In our study, 23% of resected hyperplastic EGJ lesions showed neoplastic transformation (i.e., dysplasia or adenocarcinoma), reinforcing the hypothesis that these lesions may undergo chronic irritation and regenerative proliferation, contributing to dysplastic progression 27 .

Consistent with earlier reports 6 26 28 29 , we identified three independent predictors of neoplastic transformation: non-polypoid morphology, surface ulceration, and lesion size. Notably, non-polypoid lesions were associated with substantially increased risk, mirroring findings from gastric and colorectal literature where flat or depressed lesions are more likely to harbor high-grade dysplasia or carcinoma. Surface ulceration also emerged as a significant predictor, highlighting the importance of meticulous endoscopic inspection, because ulceration may reflect more aggressive biological behavior.

Although the association between lesion size and neoplastic transformation has been previously reported 30 31 , with rates rising from 5.2% in all gastric hyperplastic polyps to 8.3% in those > 10 mm, these studies considered the entire stomach. Our study specifically addresses the EGJ, a region for which evidence remains scarce. Interestingly, GERD, BE, and *H. pylori* infection did not appear to influence neoplastic transformation in EGJ hyperplastic lesions, contrasting with what is observed in other gastric regions.

In our study, the rate of neoplastic transformation was higher than previously reported in studies that included hyperplastic lesions from the entire stomach 13 14 15 16 17 18 19 20 . This finding suggests that additional factors may contribute to development of dysplasia in the EGJ region, which presents unique characteristics such as the transition between squamous and columnar epithelium, greater exposure to gastric acid, and different patterns of *H. pylori* colonization. Our study provides a broader assessment of EGJ hyperplastic lesions, emphasizing their endoscopic characteristics and implications for early detection and resection. This supports existing recommendations to favor complete endoscopic resection of suspicious lesions, because biopsy alone is often inadequate to rule out neoplastic transformation 32 . In our series, follow-up was available for 42% of patients, and approximately 30% of resected lesions recurred, even after histologically confirmed R0 resection. This may support the theory that fibrosis and scarring contribute to new hyperplastic growth, as previously described 27 . These findings highlight the need for ongoing surveillance, even in apparently completely resected lesions.

Despite the strengths of its multicenter design and the relatively large sample size for such a specific topic, this study has several limitations. The retrospective design introduces potential selection bias, although most centers used prospectively maintained databases. A major inherent bias lies in inclusion of only lesions that underwent endoscopic resection, which were, therefore, likely considered suspicious by the endoscopist. However, biopsy alone may not provide a comprehensive assessment of dysplastic risk in EGJ hyperplastic lesions, given their potential histological heterogeneity. In addition, the macroscopic endoscopic features of these lesions are not yet clearly defined, making reliable risk characterization difficult. The other limitation is that follow-up was not standardized, and long-term outcomes were not uniformly available, limiting assessment of recurrence and progression. Furthermore, histopathological evaluation was not centralized and detailed pathological parameters were not always recorded, including information on the epithelial origin of the neoplastic proliferation (squamous versus gastric-type), thus precluding comparative analysis.

## Conclusions

In conclusion, although this retrospective multicenter study included a selected population, our findings indicate that hyperplastic lesions of the EGJ may harbor neoplastic potential, particularly when they present with suspicious endoscopic features. In such cases, especially for lesions ≥ 12 mm, ulcerated, or non-polypoid, endoscopic resection should be considered, ideally with en bloc removal to ensure complete histological assessment. Careful endoscopic evaluation and appropriate management strategies are warranted. Further prospective studies are needed to confirm the neoplastic transformation rate, validate predictive criteria, and determine whether systematic R0 resection should be recommended to reduce risk of missing neoplastic changes.

## References

[1] Bosch O González Campos CJ Esophageal inflammatory fibroid polyp - Endoscopic and radiologic features Dig Dis Sci 1994 39 2561 2566 7995180 10.1007/BF02087691

[2] Carmack SW Genta RM Schuler CM The current spectrum of gastric polyps: A 1-year national study of over 120,000 patients Am J Gastroenterol 2009 104 1524 1532 10.1038/ajg.2009.139 19491866

[3] Adorisio O Ceriati E Camassei FD Inflammatory fibroid polyp of the esophagogastric junction J Pediatr Gastroenterol Nutr 2017 64 e154 10.1097/MPG.0000000000000977 26360659

[4] Long KB Odze RD Gastroesophageal junction hyperplastic [inflammatory) polyps: A clinical and pathologic study of 46 cases Am J Surg Pathol 2011 35 1038 1044 10.1097/PAS.0b013e3182189425 21606824

[5] Kim A Park WY Shin N Cardiac mucosa at the gastroesophageal junction: An Eastern perspective World J Gastroenterol 2015 21 9126 9133 10.3748/wjg.v21.i30.9126 26290639 PMC4533044

[6] Abraham SC Singh VK Yardley JH Hyperplastic polyps of the esophagus and esophagogastric junction: Histologic and clinicopathologic findings Am J Surg Pathol 2001 25 1180 1187 10.1097/00000478-200109000-00009 11688578

[7] Murney RG Huston JD Endoscopic evaluation of the esophagogastric polyp and fold Gastrointest Endosc 1983 29 294 296 10.1016/s0016-5107(83)72636-2 6642160

[8] Melton SD Genta RM Gastric cardiac polyps: a clinicopathologic study of 330 cases Am J Surg Pathol 2010 34 1792 1797 10.1097/PAS.0b013e3181fc714d 21107084

[9] Kang MH Hoon Oh T Young Seo J Clinical factors predicting for neoplastic transformation of gastric hyperplastic polyps Korean J Gastroenterol 2011 58 184 189 22042418 10.4166/kjg.2011.58.4.184

[10] Haggitt RC Histopathology of reflux-induced esophageal and supraesophageal injuries Am J Med 2000 108 109 111 10.1016/s0002-9343(99)00346-0 10718462

[11] Zeng SX Liang YP Wu XY Gastroesophageal reflux is associated with an increased risk of gastric cardiac polyps: A case-control study of 140 cases Ann Palliat Med 2021 10 7173 7183 34154359 10.21037/apm-21-260

[12] Elhanafi S Saadi M Lou W Gastric polyps: Association with Helicobacter pylori status and the pathology of the surrounding mucosa, a cross sectional study World J Gastrointest Endosc 2015 7 995 26265993 10.4253/wjge.v7.i10.995 PMC4530333

[13] Aibo M Itabashi M Hirota T Malignant transformation of gastric hyperplastic polyps Am J Gastroenterol 1987 82 1016 1025 3661508

[14] Davaris P Petraki K Archimandritis A Mucosal hyperplastic polyps of the stomach. Do they have any potential to malignancy? Path Res Pract 1986 181 385 389 3763478 10.1016/S0344-0338(86)80072-3

[15] Ginsberg GG Al-Kawas FH Fleischer DE Gastric polyps: relationship of size and histology to cancer risk Am J Gastroenterol 1996 91 714 717 8677935

[16] Hattori T Morphological range of hyperplastic polyps and carcinomas arising in hyperplastic polyps of the stomach J Clin Pathol 1985 38 622 630 10.1136/jcp.38.6.622 4008664 PMC499259

[17] Hizawa K Fuchigami T Iida M Possible neoplastic transformation within gastric hyperplastic polyp. Application of endoscopic polypectomy Surg Endosc 1995 9 714 718 7482172 10.1007/BF00187948

[18] Zea-Iriarte WL Sekine I Itsuno M Carcinoma in gastric hyperplastic polyps. A phenotypic study Dig Dis Sci 1996 41 377 386 10.1007/BF02093832 8601386

[19] Stolte M Clinical consequences of the endoscopic diagnosis of gastric polyps Endoscopy 1995 27 32 37 10.1055/s-2007-1005629 7601032

[20] Papa A Cammarota G Tursi A Histologic types and surveillance of gastric polyps: a seven year clinico-pathological study Hepatogastroenterology 1998 45 579 582 9638455

[21] Vakil N van Zanten S Kahrilas P The Montreal Definition and Classification of Gastroesophageal Reflux Disease A Global Evidence-Based Consensus Am J Gastroenterol 2006 101 1900 1920 10.1111/j.1572-0241.2006.00630.x 16928254

[22] Endoscopic Classification Review Group Update on the Paris classification of superficial neoplastic lesions in the digestive tract Endoscopy 2005 37 570 578 10.1055/s-2005-861352 15933932

[23] Lambert R LC The Paris Endoscopic Classification of Superficial neoplastic lesions Gastrointest Endosc 2003 58 3 43 10.1016/s0016-5107(03)02159-x 14652541

[24] Nass KJ Zwager LW van der Vlugt M Novel classification for adverse events in GI endoscopy: the AGREE classification Gastrointest Endosc 2022 95 1078 85 e8 34890695 10.1016/j.gie.2021.11.038

[25] Pimental-Nunes P Libânio D Bastiaansen B A J Endoscopic submucosal dissection for superficial gastrointestinal lesions: European Society of Gastrointestinal Endoscopy [ESGE) Guideline - Update 2022 Endoscopy 2022 54 591 622 35523224 10.1055/a-1811-7025

[26] Evans JA Chandrasekhara V Chathadi KV The role of endoscopy in the management of premalignant and malignant conditions of the stomach Gastrointest Endosc 2015 82 1 8 10.1016/j.gie.2015.03.1967 25935705

[27] Imura J Hayashi S Ichikawa K Malignant transformation of hyperplastic gastric polyps: An immunohistochemical and pathological study of the changes of neoplastic phenotype Oncol Lett 2014 7 1459 1463 10.3892/ol.2014.1932 24765156 PMC3997677

[28] Forté E Petit B Walter T Risk of neoplastic change in large gastric hyperplastic polyps and recurrence after endoscopic resection Endoscopy 2020 52 444 453 10.1055/a-1117-3166 32120411

[29] Yacoub H Bibani N Sabbah M Gastric polyps: a 10-year analysis of 18,496 upper endoscopies BMC Gastroenterol 2022 22 1 7 10.1186/s12876-022-02154-8 35183117 PMC8857847

[30] Han AR Sung CO Kim KM The clinicopathological features of gastric hyperplastic polyps with neoplastic transformations: A suggestion of indication for endoscopic polypectomy Gut Liver 2009 3 271 275 20431760 10.5009/gnl.2009.3.4.271 PMC2852734

[31] Kang HM Oh TH Seo JY Clinical factors predicting for neoplastic transformation of gastric hyperplastic polyps Korean J Gastroenterol 2011 58 184 189 10.4166/kjg.2011.58.4.184 22042418

[32] Goddard AF Badreldin R Pritchard DM The management of gastric polyps Gut 2010 59 1270 1276 10.1136/gut.2009.182089 20675692

